# A Thematic Narrative Review of Osteoarthritis Risk Factors Across the Female Life Course

**DOI:** 10.3390/ijms27188083

**Published:** 2026-09-11

**Authors:** Larissa Rix, Nicola J. Kuiper, Andrew Marshall, Charlotte H. Hume, Neve O’Hare, Chris Ladel, Alzbeta Chabronova, Dharani K. Hapangama, Martyn Snow, Deborah Mason, Mandy Peffers, Karina Wright

**Affiliations:** 1 The Robert Jones and Agnes Hunt Orthopaedic Hospital, NHS Gobowen Oswestry, Liverpool SY10 7AG, UK; larissa.rix@nhs.net (L.R.); nicola.kuiper@nhs.net (N.J.K.);; 2 Centre of Science and Technology in Medicine, Keele University, Staffordshire ST5 5BG, UK; 3 Institute of Life Course and Medical Sciences, University of Liverpool, William Henry Duncan Building, Liverpool L7 8TX, UK; 4 CHL4special Consultancy, 64291 Darmstadt, Germany; 5 Liverpool Women’s Hospital NHS Trust, Liverpool L8 7SS, UK; 6 The Royal Orthopaedic Hospital, Birmingham B31 2AP, UK; 7 Arthritis UK Biomechanics and Bioengineering Research Centre, School of Biosciences, Cardiff University, Cardiff CF10 3AX, UK

**Keywords:** menopause, musculoskeletal, osteoarthritis, women’s health

## Abstract

A strong body of evidence is established that links sex-hormone-regulated physiological processes across the female life course with increased risk of osteoarthritis (OA), although the strength and nature of these associations vary by joint site and OA phenotype. Much of the available mechanistic evidence relates to knee OA with comparatively less evidence available for hip and hand OA. These include reproductive milestones (menarche and menopause), major life events such as childbirth (parity), and sex-hormone-regulated physiological functions, including menstruation and breastfeeding. Many of these have also been shown to associate with poorer surgical and non-surgical responses to musculoskeletal (MSK) treatment. A thorough multidisciplinary analysis of sex-associated differences in MSK outcomes and OA risk across the female life course is long overdue. This thematic narrative review has been conducted by a multidisciplinary team comprising experts in fundamental biology, mechanobiology, immunology, orthopaedic surgery, pain medicine and gynaecology. The review will examine in detail early life and puberty (menarche), reproductive years (menstrual cycling and injury risk, contraception, parity and breastfeeding), midlife transition (perimenopause, menopause, and treatments) and clinical interactions (response to surgical and non-surgical interventions). Biological considerations in each life-course phase/transition will focus on the hormonal milieu, biomechanical influences, immune and metabolic functions and pain pathways.

## 1. Introduction

Globally, osteoarthritis (OA) is one of the leading causes of pain and disability, representing a substantial and growing public health burden [1,2]. OA affects approximately 600 million people globally and represents a heterogeneous group of joint disorders rather than a single uniform disease entity. Knee, hip and hand OA differ in their anatomical, biomechanical and risk-factor profiles, while post-traumatic OA (PTOA) represents a secondary phenotype initiated by joint injury. Accordingly, mechanisms demonstrated at one joint site should not necessarily be assumed to apply equally across OA phenotypes [3]. The prevalence, severity, and progression of OA and responses to MSK treatments demonstrate clear sex differences, with women disproportionately affected, particularly from midlife onwards [4]. While these differences are often attributed to hormonal changes associated with menopause, emerging evidence suggests that female-specific risk factors extend across the entire life course [5]. Developmental, reproductive, and transitional stages during the life course including puberty, childbearing years, and the menopausal transition may collectively influence joint health, OA risk and clinical outcomes. To date, these stages have largely been studied in isolation, limiting our understanding of how exposures and outcomes accumulate over time [6]. A life-course perspective suggests that OA risk in women is not determined by isolated exposures but emerges from the cumulative and interacting influence of biological, hormonal, metabolic, and mechanical factors acting across successive stages of life. Developmental determinants may establish baseline susceptibility, which is subsequently modified by reproductive and hormonal events, injury and metabolic exposures, and finally by menopause-associated alterations in tissue resilience, contributing to OA development and progression.

Beyond reproductive transitions, sex differences are increasingly recognised as key determinants of MSK health and disease, influencing disease risk, clinical presentation, and treatment response. Biological, anatomical, biomechanical, and molecular factors interact throughout life to shape OA trajectories. However, sex-specific mechanisms remain largely unexplored in MSK research, contributing to persistent gaps in understanding disease pathogenesis and therapeutic outcomes. This limitation is particularly evident in the evaluation of OA interventions. For example, a recent systematic review and meta-analysis assessing intra-articular treatments for knee OA identified that only 2.5% of studies reported sex-stratified analyses, despite including over 99,000 patients, highlighting a substantial lack of sex-specific evidence [7]. Among the limited data available, no statistically significant differences in pain or functional outcomes were observed between men and women. More broadly, evidence across MSK conditions indicates that women often report greater pain severity, poorer functional outcomes, and different recovery trajectories following injury or surgery, potentially reflecting a combination of biological, biomechanical, and socio-environmental influences [4,8,9,10,11]. Structural differences, including reduced cartilage volume and thickness, as well as differences in age at presentation and baseline clinical status, have also been observed and may contribute to variation in outcomes [12,13]. Together, these findings highlight the importance of considering sex as a biological variable in MSK research and clinical practice enabling a better understanding of disease heterogeneity supporting more informed, personalised clinical decision-making.

Across the life course of both women and men, there is substantial heterogeneity in OA clinical presentation, disease progression, and responses to treatment [6]. Although the menopause has been widely studied as a key contributor to the increased burden of OA observed in women, focusing solely on this transition may overlook the cumulative influence of earlier life experiences and exposures. A life-course framework therefore offers a valuable approach for understanding how sex-specific biological and physiological factors contribute to OA susceptibility and outcomes over time. By considering these influences within a broader temporal context, it may be possible to identify critical periods for intervention and inform more personalised strategies for OA prevention and management.

## 2. Methods

### 2.1. Search Strategy

The literature was identified through searches of the electronic databases: MEDLINE (via PubMed), CINAHL Plus (via EBSCOhost), and Scopus (via Elsevier). Databases were searched from inception to July 2026. Search strategies were developed using combinations of controlled vocabulary (such as Medical Subject Headings [MeSH] and CINAHL Headings) and free-text keywords related to OA, sex differences, reproductive and hormonal factors, and key life stage transitions. No geographic or language restrictions were applied. Studies were selected based on their relevance to the review objectives, methodological quality and contribution to conceptual understanding. Priority was given to recent peer-reviewed research, ground-breaking studies and key policy documents.

### 2.2. Data Synthesis

Findings were synthesised thematically and organised within a female life-course framework, encompassing early life and puberty, the reproductive years, the menopausal transition, later life OA outcomes and treatment experiences, and broader clinical interactions, to explore how sex-specific biological and hormonal factors may influence OA risk, progression and management across the lifespan.

### 2.3. Data Reporting

Where reported, evidence was considered according to the joint site, including knee, hip and hand OA, and according to the OA phenotype. Findings derived from a specific joint site or phenotype were not assumed to be generalisable to OA as a whole.

Given that much of the epidemiological evidence relating reproductive and hormonal factors to OA is observational, associations were not interpreted as demonstrating causality. Where biological mechanisms are discussed, these are distinguished from epidemiological associations and are presented as mechanistic hypotheses unless supported by experimental or interventional evidence.

### 2.4. Terminology

Throughout this review, the terms female and male are used when referring specifically to biological sex. The terms women and men are retained where these reflect the terminology used in the cited literature or when discussing life-course experiences. Where reproductive and hormonal transitions such as menarche, menstruation, pregnancy, lactation and menopause are discussed, the available evidence predominantly relates to individuals assigned female at birth who experience these physiological processes. Evidence relating to transgender and gender-diverse populations, including the potential effects of gender-affirming hormone therapy on musculoskeletal health and OA risk, remains limited.

## 3. OA as a Sex-Dimorphic Disease

Sexual dimorphism is well established across multiple areas of medicine, where biological sex influences disease susceptibility, clinical presentation, progression and response to treatment. In cardiovascular disease, sex differences are observed in risk profiles, disease presentation, outcomes and responses to therapy, reflecting differences in hormonal, physiological and molecular mechanisms [14]. Sex also influences immune function, with females generally demonstrating stronger immune responses and a greater susceptibility to many autoimmune diseases, while susceptibility to some infectious diseases is greater in males [15]. Pharmacological responses are similarly sex-dependent, with differences in drug absorption, distribution, metabolism and elimination contributing to variation in therapeutic exposure and adverse drug reactions between females and males [16]. Collectively, these observations demonstrate that biological sex can modify disease mechanisms and clinical outcomes across diverse organ systems and provide a broader context for considering OA as a sex-dimorphic disease.

**Figure 1:**
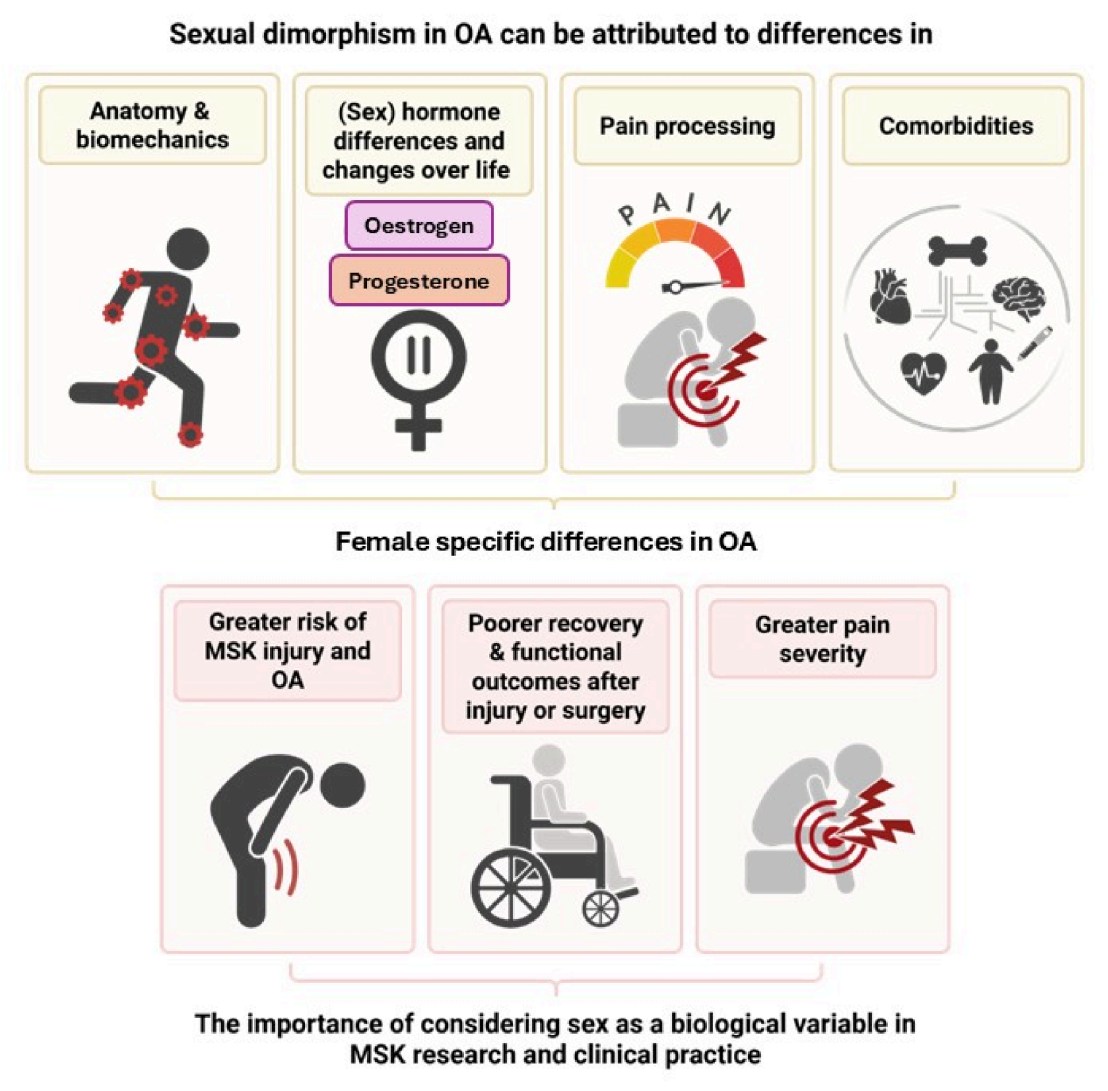
Sex dimorphisms.

Sexual dimorphism is evident across several OA phenotypes, although its magnitude and underlying mechanisms vary according to the joint site, and occurs across the life course predominantly due to differences in bone and cartilage biology, anatomy, sex hormone changes, and sex-specific differences in pain processing, muscle biomechanics, and metabolic co-morbidities (Figure 1; Table 1).

**Table 1:** Sexual dimorphism in OA across the life course.

Domain	Female Pattern	Male Pattern	Developmental Evidence	Key Sex Dimorphism	Evidence	References
Epidemiology	↑ OA prevalence following menopause; ↑ number of joint replacements.	↓ age-adjusted prevalence and more gradual age-related ↑.	Girls: ↑ ACL injuries at 2–6 times the rate of boys during adolescence. ↑ later PTOA risk.	Female OA excess emerges at menopause but appears built upon developmental and biomechanical vulnerabilities established earlier in life.	Strong	[17,18,19,20,21,22]
Cartilage Biology	↓ cartilage volume and thickness throughout life. ↑ cartilage loss after menopause.	↑ cartilage volume and thickness; ↑ matrix-maintenance gene expression.	Sex differences detectable in childhood and widen during adolescence as boys accrue cartilage more rapidly.	Females enter adulthood with ↓ cartilage reserve and distinct chondrocyte phenotype.	Moderate–strong	[23,24,25,26,27,28]
Joint Anatomy & Biomechanics	↓ femoral notch width, steeper tibial slope. ↑ laxity and knee abduction moments.	More favourable joint geometry. ↑ muscular stabilisation.	Sex-specific morphology diverges during childhood with high-risk features evident before ACL injury.	Female knee anatomy: ↑ susceptibility to ACL injury and subsequent PTOA.	Moderate	[17,25,29]
Pain Experience	↑ pain intensity, disability and sensitisation despite similar radiographic severity.	Pain correlates more closely with structural damage.	Childhood origins are unclear, though girls report poorer psychological outcomes after ACL injury.	Women experience ↑ pain burden through peripheral and central mechanisms.	Moderate	[21,30,31,32,33]
Hormonal Mechanisms	Menopause: ↓ oestrogen. ↑ inflammation and cartilage catabolism.	↑ responsiveness to androgen signalling.	Intrinsic sex differences in hormone responsiveness may precede puberty; adolescent oestrogen ↑ ligament laxity.	Female joint tissues appear hormonally primed across the life course.	Moderate	[25,34,35,36,37]
Metabolic & Co-Morbidity Profile	Menopause, chronic inflammation, diabetes and osteoporosis interact. ↑ OA progression.	Metabolic syndrome is linked to ↑ OA progression and chronic knee pain, often BMI-mediated.	Childhood BMI has limited effect on cartilage accrual; boys accrue more cartilage during adolescence.	Women may enter later life with both lower cartilage reserve and ↑ metabolic burden.	Strong	[34,38,39]
Musculoskeletal & Structural Factors	↓ muscle mass and strength, ↑ valgus alignment, ligament laxity and fatty infiltration.	↑ muscle mass and ↓ valgus loading provide protection.	Sex differences in strength and movement patterns emerge during childhood and adolescence.	Women enter midlife with ↓ muscle reserve, altered biomechanics and ↑ ligament laxity.	Moderate	[17,18,25,29,40]

Symbols: ↑, higher/increased; ↓, lower/decreased. Abbreviations: ACL, anterior cruciate ligament; BMI, body mass index; PTOA, post-traumatic osteoarthritis. Evidence-strength classification: The labels represent a qualitative synthesis of the cited literature and are not a formal GRADE assessment. Strong = consistent findings across multiple independent studies and/or complementary evidence domains; moderate–strong = generally consistent findings supported by several studies, with some heterogeneity or limitations in breadth; moderate = supportive findings, but the evidence is more limited, heterogeneous, or drawn from fewer study types. No formal risk-of-bias or certainty-of-evidence grading was undertaken.

In childhood, females have articular cartilage in the knee that is less resilient and thinner, with a biochemically different cartilage matrix, and ultimately develop an anatomy that makes them more vulnerable to injuries that lead to PTOA. Knee joint structures including the articular cartilage, bone and anterior cruciate ligament (ACL) differ between males and females in childhood [41]. In a cross-sectional MRI study of 92 healthy children aged 9–18 years, males have been reported to have 16–31% higher cartilage volume at all knee sites after adjusting for age, body mass index (BMI), bone area, and physical activity and accumulate cartilage faster during growth, specifically at sites where OA develops [23,24,42]. Young females (8–10 years) show reduced leg strength and increased joint laxity compared with boys, and these differences increase at puberty [29]. These sex-specific changes occur during a period when concentrations of the major female sex hormones, particularly oestrogen and progesterone, begin to fluctuate cyclically from menarche onwards [43,44]. Oestrogen and progesterone exert important effects on cartilage metabolism, neuromuscular control and joint laxity, and their cyclical variation may contribute to temporal changes in injury susceptibility [45]. By contrast, androgen concentrations are generally more stable over time in males, potentially leading to a different pattern of hormonal influence on both MSK development and joint health [46]. By early adolescence, girls develop significant anatomical differences in their knee compared with age-matched boys. For example, girls have smaller femoral notches, steeper lateral tibial slopes, flatter medial tibial plateaus, and shorter tibial spines [17]. Review evidence from athletic, adolescent populations indicates that females have around a 2–6-fold-higher incidence of ACL injury than males [25,47]. A systematic review and meta-analysis of nine studies examining radiographic knee OA following ACL injury, with a minimum mean follow-up of 10 years, found moderate-to-severe radiographic OA in 20.3% of ACL-injured knees compared with 4.9% of uninjured contralateral knees; ACL injury was associated with an approximately 4-fold-greater relative risk of subsequent radiographic knee OA [48]. This elevated injury risk occurs within a unique female-specific hormonal environment in which oestrogen and progesterone fluctuate, in contrast to the relatively stable androgen profile in males.

In adulthood, sexual dimorphism persists. A systematic review of 11 studies involving 1962 individuals found that men have significantly greater knee and hip cartilage volumes than women across the lifespan, with the disparity widening following the menopause [49]. Differences in knee anatomy, developed pre- and post-pubertally, continue in adulthood and increase injury risk in females, leading to PTOA [25]. Once an injury has occurred, females have a further increased risk of OA due to worse post-ACL injury outcomes including joint loading asymmetries and spatially different cartilage thickness changes [50,51].

Hormonal changes at menopause amplify each of these factors. While ovarian hormones fluctuate cyclically from menarche throughout the reproductive years, menopause is marked by a profound and sustained decline in oestrogen and progesterone levels—a transition unique to women. After 50 years of age, women consistently have higher OA incidence, increased OA severity, and higher rates of joint replacement than men [18,19]. More than 70% of women experience MSK symptoms during the menopause transition and 25% are disabled by them [18]. This is partly explained by the effects of oestrogen loss on joint tissues. The three major endogenous oestrogens are oestradiol (E2), oestrone (E1) and oestriol (E3), with 17β-oestradiol representing the most potent and biologically active form, exerting its effects primarily through oestrogen receptors ERα and ERβ, and the membrane-associated G-protein-coupled oestrogen receptor (GPER) [52]. ERα and Erβ are expressed in cartilage, synovium, and subchondral bone, and oestrogen withdrawal accelerates pro-inflammatory cytokine production, chondrocyte apoptosis, and matrix degradation [53]. Postmenopausal oestrogen loss also reduces bone mass, causing postmenopausal osteoporosis, via the Wnt/β-catenin and RANKL/OPG signalling pathways in bone [34]. These changes reflect a loss of appropriate adaptation of the bone to the mechanical environment and are likely to contribute to bone pathology in OA. Further, there are sex-specific differences in chondrocyte responses to 17β-oestradiol and chondrocyte repair processes [26,35].

The experience of pain, the major contributor to OA-related disability, is also sexually dimorphic, at least following menopause. Females undergo menopause, a unique endocrine transition characterised by profound hormonal changes that are not experienced by males [54]. Consequently, chronological age alone may not provide a biologically equivalent basis for sex comparisons in later life, and the potential confounding effects of menopause must be considered when interpreting differences in OA pain and symptoms. Menopause alters pain thresholds through central sensitisation and changes in neurotransmitter signalling, meaning that females with OA during and after menopause experience more intense and diffuse pain than men with comparable structural disease [30,55]. Further, sleep disruption from vasomotor symptoms compounds this, since poor sleep independently lowers pain thresholds [30,55].

Common conditions, such as diabetes, influence OA severity and are inherently sex-specific. Insulin resistance, obesity, and inflammatory adipokines are increased by oestrogen withdrawal in menopause, thus compounding the risk of OA in postmenopausal women. This creates a ‘metabolic hormonal’ subtype that accelerates joint degeneration disproportionately in women with diabetes [38]. Polyendocrine metabolic ovarian syndrome (PMOS), one of the most common endocrine disorders in women, is characterised by insulin resistance, obesity and hyperandrogenism [56]. PMOS may provide further insight into the relative contributions of metabolic dysfunction and altered sex hormone exposure to OA risk. For example, women with PMOS have been reported to develop OA earlier and more frequently than age-matched controls, suggesting that endocrine and metabolic factors may jointly influence joint health across the life course [57,58]. Comparisons between women with PMOS and postmenopausal women may help distinguish the effects of menopause-related hormone loss from those associated with chronic metabolic dysfunction and hyperandrogenism. In individuals with early knee OA of equivalent radiographic severity, females had greater fatty infiltration of surrounding muscles, and evidence of aberrant adipogenic reprogramming in muscle independent of age or BMI [40].

Further insight into the role of sex hormones may be gained from women exposed to markedly different endocrine environments. For example, oestrogen-containing therapies and interventions that suppress ovarian hormone production, such as gonadotropin-releasing hormone analogues or antagonists, provide informative models for understanding how both increased and reduced hormone exposure influence cartilage biology, pain pathways and OA risk across the female life course [59].

## 4. Life-Course Phases

### 4.1. Early Life and Puberty

**Figure 2:**
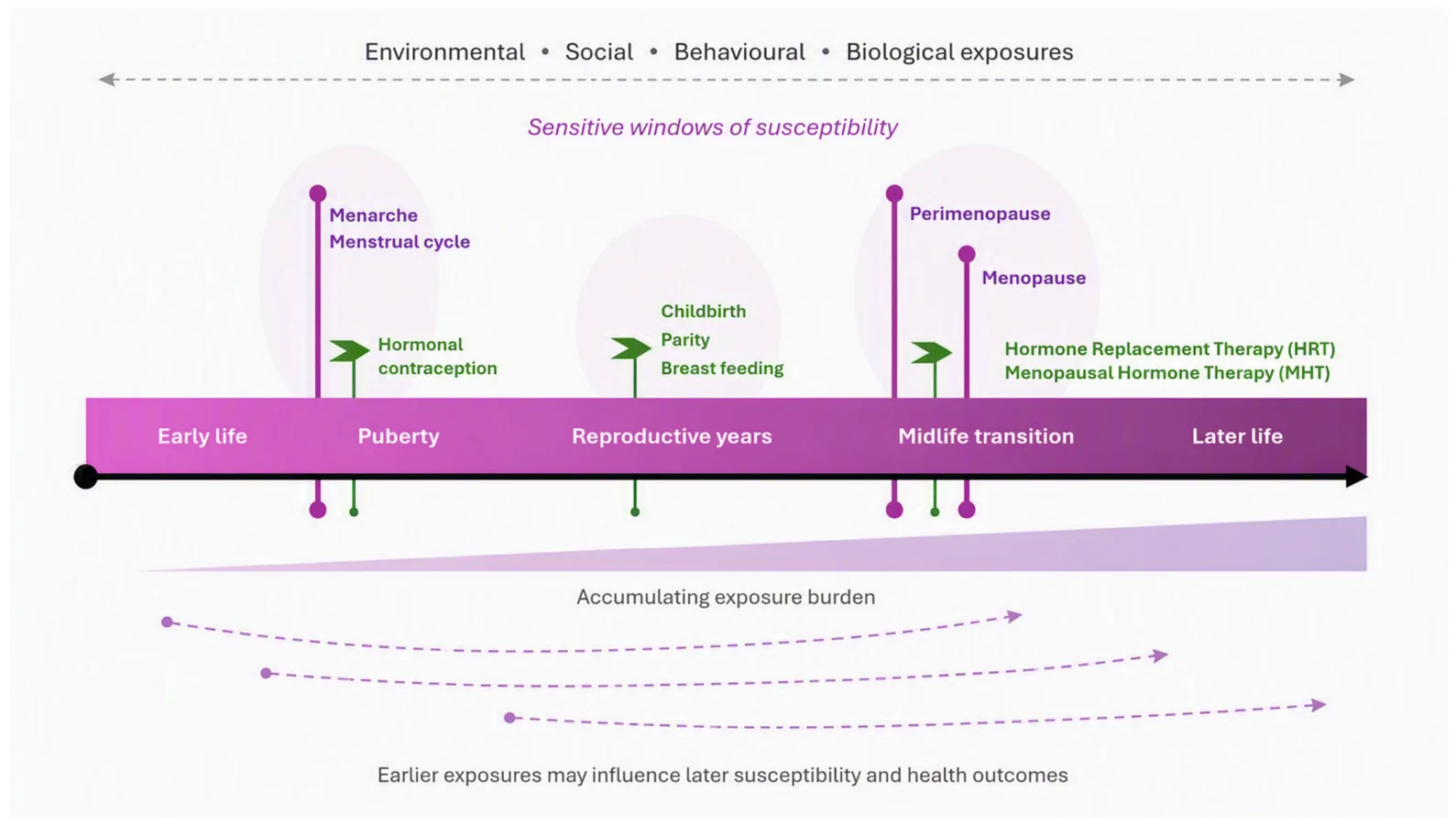
Female life-course framework illustrating the key reproductive transitions and intervention points. Environmental, social, behavioural and biological exposures may accumulate across the life course, interact during reproductive windows, and modify susceptibility to subsequent exposures, shaping health trajectories in later life.

Puberty represents the first major hormonal transition in the female life course and marks the beginning of many biological and biomechanical differences that distinguish MSK health outcomes between males and females (Figure 2). During puberty, activation of the hypothalamic–pituitary–gonadal axis results in changes to sex hormone concentrations, body composition, skeletal development and neuromuscular function [60]. Although OA is typically regarded as a disease of later life, increasing evidence suggests that pathways influencing OA susceptibility may begin during adolescence [25]. The hormonal and MSK adaptations established during puberty, together with the onset of menstrual cycling and the associated increase in injury risk, may therefore contribute to long-term joint health trajectories and represent an early window through which sex-specific OA risk emerges [61].

Menarche is defined as the onset of reproductive maturity and is a key milestone in female development. Females generally enter puberty earlier than males [62]. The rise in oestrogen production during puberty plays an essential role in bone mineral accrual, epiphyseal closure and the regulation of long bone growth, establishing many of the structural characteristics that persist into adulthood [63]. Menarche marks the onset of cyclical ovarian hormone production, establishing a distinct endocrine trajectory that persists throughout the reproductive years and differs from the more gradual hormonal changes observed in males [64]. The timing of menarche varies considerably between individuals and has been associated with several later-life health outcomes, including obesity, cardiovascular disease and MSK disorders [65]. A meta-analysis of 46 studies involving 704,869 individuals found that earlier menarche was associated with higher adult BMI and adverse cardiometabolic outcomes, supporting the concept that the timing of pubertal development may have lasting effects on health trajectories across the life course [65]. Several epidemiological studies have reported associations between earlier ages at menarche and increased risk of OA in later life [66,67]. In an observational study, Cooper et al. (1996) examined 1471 women and reported that earlier menarche was associated with increased knee OA prevalence in later life [66]. More than two decades later, in the Melbourne Collaborative Cohort Study, Hussain et al. (2018) analysed 22,289 women and found that earlier menarche was associated with increased incidence of knee arthroplasty [67]. These observational associations do not establish a direct effect of menarche on subsequent OA and, thus, the mechanisms underpinning this relationship remain uncertain. One proposed explanation is that earlier menarches are associated with increased BMI and adverse cardiometabolic characteristics across adulthood, thereby increasing cumulative mechanical loading of weight-bearing joints [65]. Additionally, socioeconomic factors, physical activity, skeletal development and the metabolic phenotype may also mediate this association. Earlier exposure to endogenous oestrogen may influence skeletal growth trajectories, growth plate maturation and joint morphology, although direct evidence linking these developmental processes to subsequent OA risk remains limited [68,69,70]. The potential influence of exogenous hormone exposure during adolescence also deserves consideration. Concerns have been raised that very-low-dose oestrogen contraceptive formulations, particularly when initiated during adolescence or early adulthood, may impair peak bone mass acquisition during a critical period of skeletal development, potentially altering lifelong MSK health trajectories [71,72]. Whether such effects extend to joint morphology, cartilage biology or later OA risk remains unknown. Consequently, age at menarche may act as a marker of a broader developmental and metabolic phenotype rather than an isolated causal determinant of OA risk.

Puberty is additionally characterised by sex-specific alterations in skeletal geometry and lower limb alignment. Females typically develop a wider pelvis relative to stature and changes in femoral and tibial alignment that may affect joint loading patterns throughout the life course [47]. While these adaptations are physiologically normal, they may contribute to biomechanical environments that influence both injury susceptibility and long-term joint degeneration. However, compared with other life-course factors, the direct contribution of menarche itself to OA risk remains relatively poorly defined. Nevertheless, increasing evidence suggests that sex-specific determinants of OA begin long before menopause, supporting the concept that puberty represents one of several critical windows during which future OA risk may be modified [25].

### 4.2. Reproductive Years

**Figure 3:**
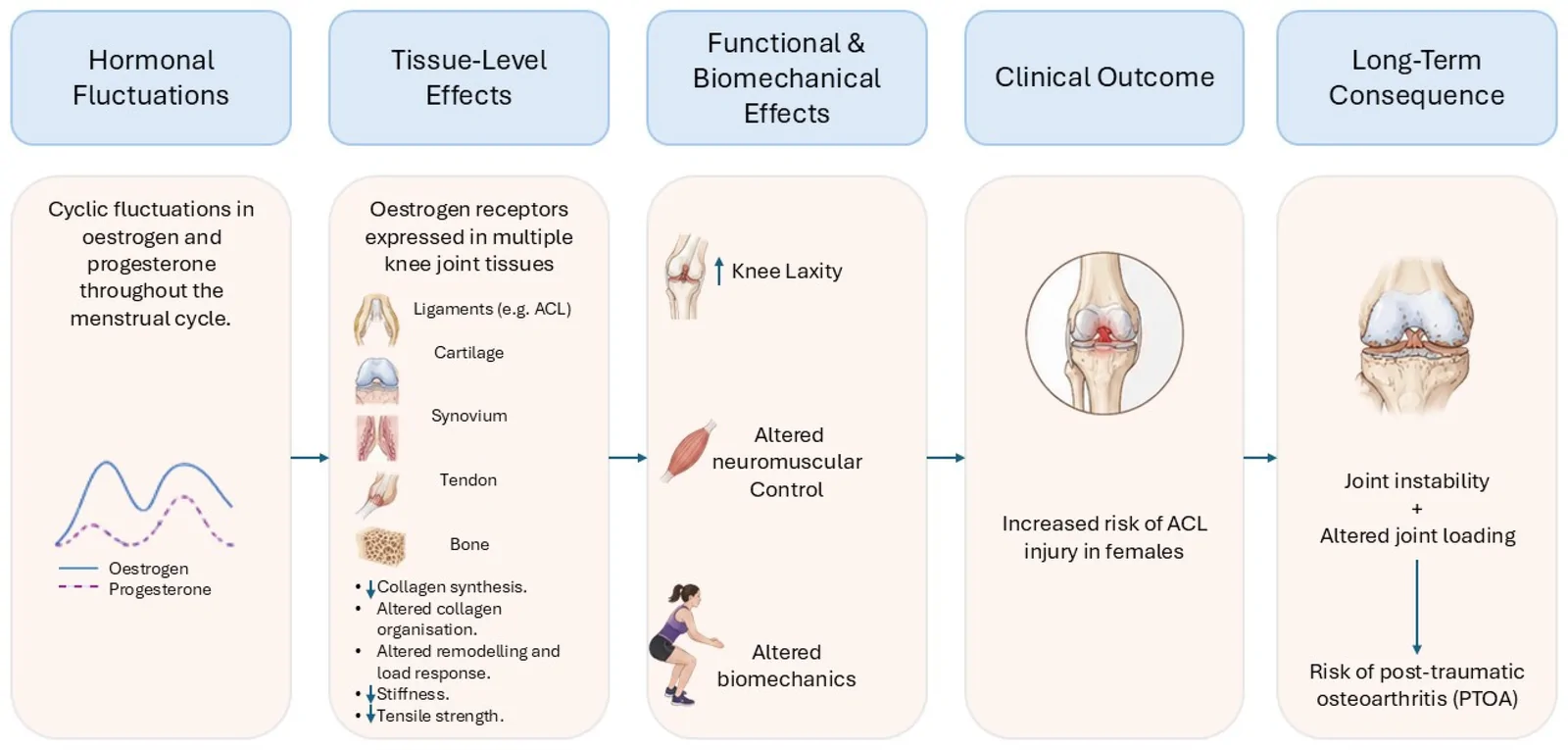
Drivers of increased ACL injury risk.

Following menarche, the start of regular menstrual cycling introduces cyclical fluctuations in oestrogen and progesterone concentrations that continue throughout the reproductive years. These hormonal variations exert effects beyond reproductive tissues, influencing ligaments, tendons, skeletal muscle, bone and the nervous system (Figure 3) [73]. As a result, considerable interest has focused on whether menstrual cycle-related hormonal changes contribute to the increased incidence of MSK injuries observed in females.

Given the established association between ACL injury and subsequent knee OA, it is notable that sex differences in ACL injury risk emerge during adolescence following pubertal maturation [47]. This observation has prompted extensive investigation into the role of sex hormones in injury susceptibility. Experimental studies suggest that elevated oestrogen concentrations may reduce collagen synthesis, alter collagen organisation and affect tissue mechanical properties, which may influence ligament stiffness and tensile strength, potentially affecting the capacity of the ACL to withstand dynamic loading [73]. Fluctuations in joint laxity provide further evidence for hormone-mediated changes in MSK function. Multiple studies have demonstrated variations in anterior knee laxity across the menstrual cycle, with some women exhibiting increased laxity during phases associated with elevated oestrogen concentrations [74,75]. In a longitudinal study, Perrin et al. (2005) followed 22 healthy women across a complete menstrual cycle and demonstrated an association between hormonal fluctuations and increased knee laxity [74]. Similar findings were reported by Shultz et al. (2010) in an independent cohort of 66 naturally menstruating women, confirming that cyclic changes in ligament laxity occur in a proportion of naturally menstruating females [75]. Increased laxity may alter knee biomechanics, increasing anterior tibial translation and placing greater strain on the knee during landing and pivoting movements, contributing to the higher incidence of non-contact injuries observed in female athletes. These biomechanical changes are important as ACL rupture is one of the strongest established risk factors for premature knee PTOA [76].

From a knee OA perspective, the significance of menstrual cycle-associated injury risk lies in the long-term consequences of joint trauma. Given the established association between ACL injury and later joint degeneration, it is noteworthy that a systematic review and meta-analysis of nine studies (*n* = 1061) found that approximately 20% of individuals develop moderate/severe radiographic OA within 10 years of injury, regardless of reconstruction status [48]. The mechanisms are multifactorial and include altered joint biomechanics, persistent instability, cartilage injury, meniscal damage and chronic low-grade inflammation. Importantly, ACL injuries often occur during adolescence and early adulthood, decades before the onset of symptomatic knee OA. This creates a plausible life-course pathway linking female reproductive physiology to later joint degeneration. While menstrual cycling itself is unlikely to directly cause knee OA, hormonal influences on ligament properties, neuromuscular control and injury susceptibility may increase exposure to joint trauma, thereby contributing indirectly to OA risk [48,73,77]. This relationship may be particularly relevant in physically active populations where ACL injury incidence is highest. Emerging evidence also proposes that hormonal status may influence recovery following MSK injury. Oestrogen has recognised effects on inflammation, collagen turnover and tissue healing, raising the possibility that hormonal fluctuations could influence post-injury knee joint remodelling and subsequent OA progression [73]. However, this remains an area requiring further investigation.

Hormonal influences are not restricted to passive tissue properties. Ovarian hormones also affect neuromuscular control through actions on the central and peripheral nervous systems. A systematic review evaluated available evidence from 17 studies and concluded that the menstrual cycle phase may influence lower limb biomechanics, proprioception and neuromuscular control; however, findings remain inconsistent due to methodological heterogeneity [77]. Altered recruitment of the hamstrings and gluteal musculature, combined with increased dynamic knee valgus, has been proposed as a mechanism contributing to injury risk in females [47,77]. These neuromuscular factors may interact with anatomical and biomechanical characteristics established during puberty, further influencing injury susceptibility. One of the earliest studies to investigate this relationship analysed 28 female athletes with ACL injuries and reported a greater proportion of injuries occurring during the ovulatory phase of the menstrual cycle [78]. Although subsequent studies produced conflicting findings, this work stimulated extensive investigation into hormonal contributions to ACL injury risk [78]. Despite compelling mechanistic evidence, attempts to identify a specific menstrual cycle phase associated with heightened injury risk have yielded inconsistent findings. Some studies have suggested increased injury risk during the ovulatory phase when oestrogen concentrations peak, while others have implicated the pre-ovulatory or luteal phases [79]. A recent systematic review which included seven studies of naturally menstruating women concluded that substantial methodological heterogeneity, including inconsistent cycle verification methods and small sample sizes, currently limits definitive conclusions regarding the existence of a distinct high-risk phase [79].

Consequently, although hormonal fluctuations appear to influence several recognised injury risk factors, the precise relationship between the menstrual cycle phase and ACL injury remains incompletely understood. These inconsistencies may also help explain the conflicting literature regarding hormonal contraceptive use and OA risk. Unlike naturally menstruating women, users of hormonal contraceptives experience altered endogenous hormone profiles and reduced cyclical fluctuations in oestrogen concentrations. Consequently, the effects of hormonal exposure on joint tissues, neuromuscular control and injury risk may differ substantially between populations, making direct comparisons challenging and potentially contributing to the contradictory findings reported in the OA literature. The elevated risk of ACL injury in female athletes, particularly those participating in endurance and weight-sensitive sports, may be further exacerbated in those with menstrual dysfunction, including amenorrhoea, where low energy availability, hormonal suppression and impaired neuromuscular control combine to increase susceptibility to knee injury and could indirectly influence later OA risk through joint injury, although evidence remains limited [80,81,82,83].

As well as the increased injury risk described, several significant life events may occur in the years between menarche and menopause, all of which may influence OA risk. Pregnancy and lactation represent additional female-specific physiological transitions that may influence MSK health [84]. Pregnancy is characterised by significant endocrine and metabolic adaptations, followed by a rapid postpartum hormonal shift, while lactation is associated with increased calcium demands and transient reductions in bone mineral density, which generally recover following weaning. Pregnancy also induces substantial biomechanical changes, including altered joint loading and increased ligamentous laxity [85]. Consequently, pregnancy and lactation should be considered important life-course exposures that may shape long-term MSK health and contribute to sex differences in ageing-related MSK disorders.

Parity, breastfeeding and the use of oral contraception represent distinct hormonal changes associated with hormonal, biomechanical and metabolic drivers of OA; however, unpicking the role of each of these with subsequent OA is predominantly observational. The dose–response signal for parity is the most frequently replicated finding in this body of work. In the longitudinal observational ‘Multicentre Osteoarthritis Study’ (MOST) cohort of 1618 women aged 50–79 years with, or at increased risk of, knee OA, women reporting to have had five or more births had a 2.6-fold-higher adjusted risk of incident radiographic knee OA and a 2.7-fold increase in knee replacements over 30 months, compared to women reporting one birth, and the Million Women Study (n = 1.3 million) and the Danish national register (n = 4.6 million) both reproduced a positive association between each additional live birth and OA, with a stronger effect at the knee than the hip [86,87,88]. Additionally, the Women’s Health Initiative sub-study (n = 130,331 parous women) demonstrated that a history of parity was statistically associated with OA; however, this was not clinically significant [89]. Interestingly, the Danish cohort found the parity–OA association was similar or stronger in men than women, which is difficult to explain through a maternal hormonal pathway and points instead toward a shared maternal endocrine mechanism related to child rearing, socioeconomics and altered biomechanics associated with the loading of carrying children [88]. The particularly strong association between parity and knee OA in women nevertheless leaves open the possibility of additional pregnancy-specific contributions.

Potential pregnancy-specific mechanisms therefore remain hypotheses rather than established causal pathways. Studies to test the biomechanical consequences of pregnancy or parity-associated weight gain during pregnancy have been very limited. The 2022 Finnish birth cohort study found gravidity (the number of pregnancies, not the number of foetuses or babies born) and parity were associated with increased tibial plateau and femoral condylar breadth at age 46 and that each pregnancy was associated with 0.11–0.14% larger knee breadth, indicating that repeated gestational weight-bearing may permanently remodel joint geometry [90]. A small gait study demonstrated altered knee extension and hip adduction moments in parous versus nulliparous women, together indicating that there is a coherent (if underpowered) mechanical hypothesis contributing to parity increasing OA risk in weight-bearing joints and highlighting that the contributions of hormonal and biomechanical drivers require unravelling [91].

For women who have had children, breastfeeding introduces another significant hormonal change, with lactation inducing a discrete, sustained hypoestrogenic state [92]. Changes in oestradiol during lactation have been referred to as a state analogous to menopause; therefore, exposure to some of the mechanisms underlying menopause-driven OA may occur during this period [93]. Pregnancy itself represents a distinct endocrine and biomechanical exposure. Marked increases in relaxin, oestrogen and progesterone concentrations alter collagen turnover and connective tissue physiology, contributing to the increased ligamentous laxity observed during pregnancy. Concurrent increases in body mass, changes in posture and gait, and altered patterns of joint loading further modify the mechanical environment of weight-bearing joints. Although many of these adaptations are reversible following delivery and weaning, repeated exposure across multiple pregnancies, or incomplete recovery of MSK tissues, may have implications for long-term joint stability. Studies assessing associations between lactation and OA provide consistent results, although the phenotypes assessed differ between studies and the most specific radiographic evidence relates to knee OA. Three independent observational studies, one in the USA (n = 1769) and two Korean (n = 10,102; n = 3454), have reported associations in the direction and dose–response of breastfeeding on OA: longer breastfeeding duration, and a higher number of children breastfed, both independently increase OA risk [94,95,96]. Since these studies compared women of similar age and demonstrated a dose–response relationship after adjustment for key confounders, differential survival alone is unlikely to account for the observed associations. Whilst Park et al. (2017) and Ham and Bae (2023) relied on self-reported OA diagnosis, greater confidence in the relationship between breastfeeding and OA is supported when considered alongside Kim et al. (2020), where radiographic assessment (Kellgren Lawrence Grades 2–4) reduced reliance on self-reported OA diagnosis [94,95,96]. One potential mechanistic hypothesis explaining the relationship between the number of children breastfed and increased OA risk is that repeated or prolonged exposure to a low-oestrogen state across multiple pregnancies may have a cumulative effect on cartilage and subchondral bone, mirroring the established oestrogen deficiency mechanism in postmenopausal OA [97]. However, the available epidemiological studies do not demonstrate that lactation-associated hypoestrogenism mediates the observed association, and confounding by parity, BMI, socioeconomic factors, physical activity and other reproductive characteristics remains possible.

The extent to which these associations also apply to hip, hand or other OA phenotypes remains unclear. The mechanisms underlying breastfeeding and OA are highly underexplored; the vast majority of all OA–hormone research has focused on the menopausal transition as the single critical hypoestrogenic event, while breastfeeding represents repeated, potentially much earlier hypoestrogenic episodes that current studies do not account for.

Oral contraception introduces another interesting hormonal influencer during reproductive years. Globally, 151 million women are estimated to rely on oral contraception, marking a dramatic change in family planning for women over the past 60 years [98]. Importantly, oral contraceptives are not a homogeneous exposure. They broadly comprise combined oral contraceptives (COCs), which contain both oestrogen and progestogen and generally maintain circulating oestradiol levels, and progestogen-only pills (POPs), which can induce amenorrhoea and ovarian suppression in approximately 40% of women, resulting in lower circulating oestradiol levels [99,100]. Consequently, these formulations may have differing effects on joint tissues and OA risk, and combining all oral contraceptive users into a single exposure category may obscure important biological differences. The literature assessing oral contraception and OA risk is highly geographically fractured. A prospective UK study of 1.3 million women demonstrated no association between oral contraceptive use and hip or knee joint replacement risk [87]. Contrastingly, a nationwide study of 1.13 million women in Korea found that oral contraceptive use was associated with increased risk of knee arthroplasty (regardless of time using oral contraception) and hip arthroplasty (if oral contraceptives were used for >1 year) [101]. In another Asian cross-sectional cohort of 7510 women in China, oral contraceptive use independently increased the odds of knee pain and radiographic OA [102]. Interestingly, this study also reported an additive interaction between oral contraception use and BMI, such that women with both elevated BMI and a history of oral contraceptive use had roughly double the odds of knee pain compared to women with neither factor [102]. These observational findings cannot establish a causal effect of oral contraception on OA, particularly because contraceptive exposure is heterogeneous and may correlate with reproductive history, BMI, metabolic status, healthcare utilisation and other behavioural or socioeconomic factors. There are multiple plausible explanations for the differences across studies, including population-level differences in baseline BMI and metabolic risk; differences in ethnicities across the studies; or that oral contraceptive formulations and doses vary across the regions or the time periods studied. Further studies are needed to unpick whether BMI and oral contraceptive interactions are retained across diverse populations and whether associations with OA differ according to contraceptive formulation and resultant hormonal milieu.

Overall, the literature suggests that the shared hormonal window of menarche to menopause is shaped not by a single exposure but by overlapping events, menstrual cycling, parity, lactation and oral contraception, each with distinct and sometimes conflicting associations with OA risk. Disentangling hormonal from biomechanical and metabolic contributions, particularly BMI interactions, across diverse populations remains essential to clarifying female-specific OA pathways.

### 4.3. Midlife Transition

Perimenopause is the transitional period leading up to menopause, during which ovarian function declines and hormone levels fluctuate. Menopause is defined as the permanent cessation of menstruation following 12 consecutive months without a menstrual period [103,104]. More than 47 million women worldwide enter menopause each year, and this stage of life is associated with a range of MSK changes that can affect joint health, pain, and physical function [105]. OA becomes increasingly prevalent in women during midlife. Incidence rates rise sharply around the menopausal transition, and women experience a greater burden of OA symptoms, including pain, joint stiffness and functional limitations [106]. Epidemiological evidence indicates that OA is more prevalent in postmenopausal women than in both premenopausal women and men of a similar age [101,106,107]. The association between shorter lifetime exposure to oestrogen and an increased risk of severe knee OA in postmenopausal women provides further support for a role of hormonal factors beyond chronological ageing alone [101].

During perimenopause, oestrogen levels fluctuate before gradually declining [103,107]. Experimental evidence suggests that oestradiol mitigates stress-induced chondrocyte death, potentially contributing to protection against OA cartilage degeneration [34,108]. Consequently, reduced oestrogen availability may increase tissue vulnerability and contribute to the initiation and progression of OA [34,108]. Oestrogen deficiency can interfere with normal cartilage maintenance, promote low-grade inflammation, and alter metabolic processes within joint tissues, increasing OA risk [108,109,110]. In addition, hormonal changes during the menopausal transition are often associated with increased body weight and reduced muscle strength [53,111]. These changes may increase biomechanical loading on weight-bearing joints, particularly the knee, while reduced muscle strength may compromise joint stability and further accelerate joint degeneration [19,112].

Further support for the importance of ovarian hormones in MSK ageing may be derived from women with premature ovarian insufficiency or surgical menopause, who experience an earlier and often more abrupt decline in ovarian function and oestrogen exposure than occurs during natural menopause [113]. Whilst the adverse effects of these conditions on bone health and osteoporosis risk are well established, their consequences for cartilage biology, joint health and OA development remain less clearly defined. Nevertheless, these populations provide important models for investigating the effects of prolonged oestrogen deficiency and the potential modifying effects of hormone replacement therapy (HRT) on MSK ageing.

In preclinical models altered oestrogen receptor expression was observed in OA cartilage, with reduced ERα expression compared with healthy tissue, suggesting impaired oestrogen signalling may contribute to cartilage degeneration [114]. Similarly, menopause has been associated with altered synovial protein abundance in patients with OA, and a loss of bone mass, providing further evidence that joint tissues are responsive to changes in oestrogen status [115,116]. Experimental studies with ovariectomised animal models demonstrated accelerated cartilage degeneration and adverse subchondral bone changes following oestrogen deficiency [117]. Further studies found restoring oestrogen signalling reduced structural damage and improved joint integrity [118,119]. Together, these findings suggest that declining oestrogen during menopause may contribute to OA pathogenesis through effects on cartilage homeostasis and bone remodelling [117,118,119].

Taken together, these experimental findings may provide biological plausibility for a direct effect of oestrogen deficiency on joint tissues. However, they should be distinguished from the human epidemiological evidence, in which menopause and lower lifetime oestrogen exposure are associated with OA outcomes but remain closely correlated with age, adiposity, metabolic change, physical activity and other features of midlife ageing. Consequently, whilst declining oestrogen represents a plausible contributor to OA pathogenesis, the causal contribution to human OA development remains unresolved.

Fluctuating and declining oestrogen levels may also affect pain perception during perimenopause [103]. Findings suggest that reductions in oestrogen are associated with increased MSK pain sensitivity and contribute to the development of joint symptoms during the menopausal transition [31,120,121,122]. These symptoms are associated with reduced physical function and a lower quality of life [18]. Declining oestrogen may contribute to these symptoms by increasing pain sensitivity through oestrogen receptors within the peripheral and central nervous systems (Table 2) [18,31]. Furthermore, declining oestrogen promotes a pro-inflammatory environment characterised by increased production of cytokines such as interleukin 6 (IL-6) and tumour necrosis factor alpha (TNF-α). Consistent with this, postmenopausal women with knee OA have been reported to exhibit lower oestradiol oestrogen levels alongside elevated inflammatory markers, including C-reactive protein and IL-6 [123]. Changes in other reproductive hormones, including rising follicle-stimulating hormone (FSH) and progesterone may also influence inflammatory pathways and pain perception, although their precise roles are less well established [124]. These biological changes may help explain the increased frequency of joint pain, stiffness and functional limitations reported by many women during and after the menopause transition [18].

Given the apparent importance of oestrogen in maintaining joint health, HRT has been investigated as a potential modifier of OA risk and progression [88,89]. Several observational studies have reported that HRT is associated with a reduced risk of hand and spinal OA, particularly when treatment is initiated around the menopausal transition and maintained long-term [125,126,127]. This is because declining oestrogen levels, together with fluctuations in progesterone and an increase in FSH, during menopause may contribute to MSK symptoms. However, these potential protective effects are not consistently observed across all populations in all joint sites. Some studies report no association between HRT use and OA or hand OA symptoms [128,129,130] whereas other studies have reported an increased risk of knee OA [131]. Greater cartilage loss after menopause may contribute to the increased risk of OA in women [49]. This demonstrates that the effects of HRT may differ according to the joint site, timing of therapy and duration of treatment [131].

**Table 2:** Clinical and experimental evidence supporting a role for menopause-associated oestrogen deficiency in osteoarthritis development and progression.

Menopause-Related	Physiological Domain	Study Type	Key Findings	References
OA Risk	Epidemiology/reproductive factors	Human in vivo—cohort	Shorter lifetime exposure to oestrogen was associated with a greater risk of knee and hip replacement because of OA.	[112]
OA Risk	OA prevalence	Human in vivo—population study	Postmenopausal women had a higher prevalence of clinical OA at the hip, knee and hand.	[19]
OA Risk	OA epidemiology	Human in vivo—prospective study	Demonstrated increasing incidence of hip and knee OA in middle-aged women, supporting increased OA after menopause.	[101]
OA Risk	Reproductive factors/OA	Human population-based observational study	Reproductive factors were associated with OA risk, with menopause and BMI contributing to the relationship between reproductive history and OA.	[132]
OA Risk	OA epidemiology/menopausal risk factors	Human registry-based observational study	Identified menopause-related and metabolic risk factors associated with OA in menopausal women, supporting menopause as an independent contributor to OA development.	[133]
OA Risk	Hormone exposure/OA risk	Human in vivo—retrospective population study	Lifetime hormone exposure following menopause was associated with OA risk, supporting a relationship between declining oestrogen levels and OA development.	[134]
Musculoskeletal Symptoms	Musculoskeletal pain	Human in vivo—observational study	Musculoskeletal pain increased after menopause and was associated with menopausal status.	[53]
Musculoskeletal Symptoms	Menopausal symptoms	Human in vivo—longitudinal study	Hormonal changes during the menopausal transition were associated with increasing joint pains and aches.	[135]
Musculoskeletal Symptoms	Pain during menopause	Human in vivo—longitudinal study	Pain symptoms increased across the menopausal transition and persisted into postmenopause.	[115]
Cellular Targets	Cartilage	Human in vitro	Identified oestrogen receptors in human articular chondrocytes, indicating cartilage responds directly to oestrogen.	[136]
Cellular Targets	Synovium	Human in vitro	Synovial tissue contains multiple oestrogen receptor subtypes, suggesting declining oestrogen may alter synovial function and contribute to OA.	[125]
Cellular Targets	Oestrogen receptors/cartilage	Human ex vivo tissue study	Oestrogen receptor-α was identified in healthy and osteoarthritic human knee cartilage, supporting a role for oestrogen signalling in cartilage homeostasis.	[137]
Cellular Targets	Bone (osteoblasts)	Human in vitro	Female osteoarthritic osteoblasts expressed oestrogen receptor-α (ERα) and demonstrated a distinct biological response compared to male osteoblasts, supporting sex-specific regulation of bone remodelling by oestrogen signalling.	[36]
Joint Tissue Degeneration	Cartilage/subchondral bone	Mouse in vivo—animal study	Oestrogen deficiency accelerated cartilage degeneration and abnormal subchondral bone remodelling, while oestrogen protected joint tissues.	[138]
Joint Tissue Degeneration	Oestrogen signalling/cartilage	Mouse in vivo—animal study	Impaired oestrogen signalling increased cartilage degeneration and OA severity, supporting a protective role of oestrogen in maintaining joint integrity.	[139]
Joint Tissue Degeneration	Cartilage/subchondral bone	Mouse in vivo—animal study	Oestrogen deficiency promoted cartilage degeneration and abnormal subchondral bone remodelling, demonstrating that oestrogen regulates joint tissue homeostasis.	[140]
Joint Tissue Degeneration	Oestrogen receptor modulation/cartilage	Mouse in vivo—animal study	Ovariectomy-induced oestrogen deficiency caused cartilage degeneration and subchondral bone deterioration, while raloxifene attenuated these changes.	[141]
Joint Tissue Degeneration	Bone/osteoblast	Mouse in vivo—animal study	Osteoblast-specific deletion of ERα reduced bone mass and increased the severity of load-induced osteoarthritis in female mice, demonstrating that impaired oestrogen signalling in bone contributes to OA progression through altered subchondral bone remodelling.	[142]
Inflammation and Synovial Changes Following Menopause	Musculoskeletal	Human observational laboratory-based study	Menopause was associated with altered synovial tissue protein expression in women with knee OA.	[143]
Inflammation	Inflammation/oestradiol	Human in vivo—observational study	Lower circulating oestradiol levels were associated with increased inflammatory markers in postmenopausal women with knee OA.	[142]
Inflammation	Inflammation/oestrogen	Human in vivo—case–control study	Elevated IL-6 and hs-CRP were associated with symptomatic lumbar OA in postmenopausal women, supporting an inflammatory mechanism.	[144]
Therapies	Cartilage/oestrogen deficiency	Rat in vivo—animal study	S-equol reduced oxidative stress, cartilage degeneration and inflammation in ovariectomised rats, suggesting potential therapeutic benefit.	[145]
Therapies	Hand OA/perimenopause	Human pilot clinical study	Equol supplementation improved pain and hand function in perimenopausal women with hand OA.	[146]
Therapies	Hand OA/oestrogen-related factors	Human in vivo—observational study	Equol production capability and family history were associated with hand OA risk, suggesting oestrogen-related metabolic factors influence susceptibility.	[147]

IL-6, interleukin-6; hs-CRP, high-sensitivity C-reactive protein; S-equol, biologically active form of equol (phytoestrogen).

The inconsistencies reported in the literature may reflect differences in study design (Table 3). Several studies have suggested that HRT may reduce the risk or severity of OA, whereas others have reported little or no clear protective association [127,128,129,130,131,148]. Most of the evidence available is observational. Consequently, it is difficult to determine whether HRT directly influences OA risk or whether observed associations are attributable to confounding factors such as age, body weight, overall health status, lifestyle factors, or levels of systemic inflammation [149]. Furthermore, substantial heterogeneity exists across studies in terms of HRT formulation, the timing of initiation, dosage, and duration of use [126,127,130]. Given the predominance of observational evidence, causality cannot be established, and the current body of research remains insufficient to draw definitive conclusions regarding the effectiveness of HRT in preventing OA onset or slowing disease progression [19,53,149]. In conclusion, the menopausal transition may play an important role in OA development and progression through hormonal changes, particularly declining oestrogen levels. However, uncertainty surrounding the underlying mechanisms and the effect of HRT highlights the need for high-quality, female-specific research adopting a life-course perspective to better understand OA pathogenesis and inform prevention and treatment strategies [110].

### 4.4. Later Life: OA Pain/Sensitisation and Pharmacological Treatments

**Figure 4:**
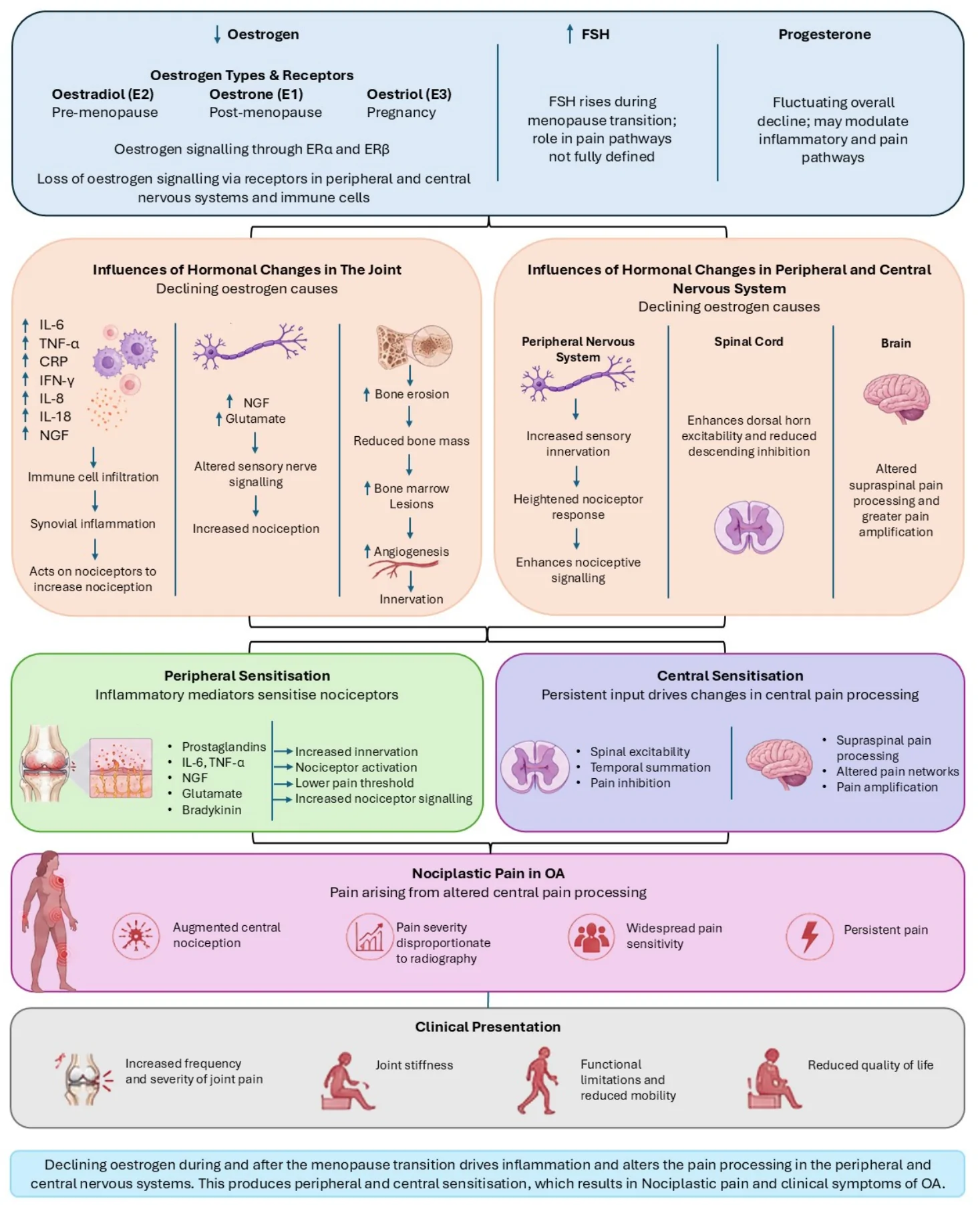
Drivers of pain in the menopause.

An association between OA pain onset or worsening and menopause (“menopausal arthralgia”) has been recognised for over a century [154,155]. Mechanisms described earlier in terms of oestrogen loss persist in later life; however, differences in OA pain in women are increasingly recognised as arising from a complex interplay between peripheral inflammation, peripheral and central sensitisation, as well as hormonal influences (Figure 4) [156,157,158]. Pain originates in innervated joint tissues, where inflammatory mediators include prostaglandins, IL-6, TNF-α, and nerve growth factors, together with excitatory neurotransmitters such as glutamate, sensitise peripheral nociceptors and increase nociceptive signalling [157,159,160,161]. Osteoclasts facilitate innervation of the joint and the presence of bone marrow lesions, or remodelled bones are the only structural joint changes associated with OA pain [162]. In a study of 818 postmenopausal women, treatment with the bone-sparing bisphosphonate, alendronate, and oestrogen decreased the prevalence of knee OA-related subchondral bone marrow lesions and was associated with reduced knee pain [163]. Persistent peripheral input can drive central sensitisation, resulting in enhanced excitability of spinal and supraspinal nociceptive pathways [157,159]. Consequently, many patients with OA exhibit features of nociplastic pain, pain arising from altered central pain processing rather than ongoing tissue damage including augmented central nociception, widespread pain sensitivity, and pain severity disproportionate to radiographic findings [55,164,165,166].

Women exhibit greater pain sensitivity than men across numerous chronic pain conditions, including OA. These differences are influenced by interactions among sex hormones, immune responses, and central and peripheral pain pathways [167,168,169,170]. Sex-specific inflammatory profiles may also contribute to female-specific OA pain. Compared with men with OA, women exhibit higher serum C-reactive protein levels, and studies have reported increased concentrations of pro-inflammatory cytokines in serum, synovial fluid, and cerebrospinal fluid, including interferon (IFN)-γ, IL-3, IL-8, IL-18, TNF-β, and other macrophage-stimulating factors [36,171,172].

Understanding the neurobiology of OA pain in women has important therapeutic implications. Management strategies should extend beyond structural joint preservation to target neuroinflammation, central sensitisation, and other mechanisms underlying nociplastic pain. Incorporating menopausal status into clinical decision-making may facilitate more personalised, sex-specific pain management. Future studies employing female-specific preclinical models and longitudinal clinical cohorts are needed to elucidate interactions between menopause, neuroimmune signalling, and central pain processing to identify novel therapeutic targets that reduce the disproportionate burden of OA pain in women. Furthermore, future research should adhere to emerging recommendations for the rigorous inclusion and analysis of sex and gender as biological and sociocultural variables, thereby improving the reproducibility, translatability, and clinical relevance of OA pain research [173].

International guidelines recommend exercise, weight management, and education as first-line interventions across common OA phenotypes, although individual recommendations differ between knee, hip and hand OA, supported by moderate-to-high-quality evidence demonstrating improvements in pain and physical function [174,175,176]. However, treatment response varies considerably, and little research has examined whether sex, menopausal status, or endocrine factors influence therapeutic efficacy. Pharmacological treatment is similarly directed towards symptom control, with topical and oral non-steroidal anti-inflammatory drugs (NSAIDs) providing the greatest analgesic benefit, while intra-articular corticosteroids offer only short-term relief and are unsuitable for repeated long-term use because of concerns regarding cartilage toxicity [174,177].

No current pharmacological interventions halt structural disease progression or target the biological mechanisms underlying OA. Furthermore, repeated failures of disease-modifying osteoarthritis drugs (DMOADs) in late-stage clinical trials highlight the limited translation of promising preclinical discoveries [178,179]. A major contributor may be the continued reliance on experimental models that inadequately represent female biology. Although women comprise most individuals with symptomatic OA, preclinical studies continue to preferentially use young male animals or fail to report sex as a biological variable [180]. Reproductive ageing, menopause, and oestrogen deficiency are rarely incorporated into study designs despite their established effects on joint tissues, inflammation, and pain pathways. Incorporating female-specific, hormonally relevant preclinical models is essential to improve biomarker discovery, identify sex-specific therapeutic targets, and enhance clinical translation for the population most affected by OA.

## 5. Differential MSK Surgical Outcomes

Sex is increasingly recognised as an important factor influencing MSK surgical outcomes. For patients with advanced disease unresponsive to conservative management, total joint arthroplasty remains the definitive treatment, substantially improving pain, function, and quality of life [181]. Nevertheless, women typically undergo surgery with greater pain, disability, and functional impairment than men and, despite comparable implant survival, often report less favourable postoperative outcomes [182,183,184]. In a large retrospective cohort of over 6 million hip and knee arthroplasty patients between 2022 and 2011, complication rates varied by sex, with no consistent overall disadvantage observed for either group [185]. Sex-based differences in arthroplasty outcomes indicate that women experience greater postoperative pain compared to men [186]. Taken together, these findings suggest that although women often present with worse pre-operative status, they achieve comparable relative improvements following surgery but may follow different recovery trajectories and not reach the same postoperative outcome levels as men [187]. This pattern is consistent across other MSK procedures. Women undergoing meniscal surgery report reduced functional scores and quality of life compared to men, despite achieving similar improvements in knee outcomes [188]. Likewise, in a large registry study of 4986 patients undergoing cartilage repair, women had worse baseline and postoperative functional scores but demonstrated greater relative improvements in function; however, they reported lower satisfaction and higher rates of further surgery [13]. These outcome differences likely reflect disparities present at the time of surgical presentation. Across MSK conditions, women have worse baseline patient-reported outcomes, including greater pain and functional limitation, compared with men [189]. Importantly, these differences persist after adjustment for objective measures of disease severity—such as radiographic grade in OA or tear size in rotator cuff disease indicating a higher symptom burden despite similar disease severity in women. This suggests that sex-related differences in pain processing, psychosocial factors, and healthcare access may contribute to delayed presentation and increased symptom burden at the time of surgery [190].

More marked sex differences appear to occur following MSK procedures that have complex recovery pathways. In ACL reconstruction, a systematic review and meta-analysis of self-reported activity and knee-related outcomes show women have inferior self-reported activity and return-to-sport outcomes [21]. Similarly, in rotator cuff surgery, women consistently presented with worse pain, poorer functional status, and reported a different experience in their management and recovery compared to men [9]. Following spine surgery, women are more likely to experience wound-related complications, prolonged length of stay and non-routine discharge [191]. Women undergoing MSK surgery often present with a higher burden of co-existing long-term conditions, including cardiovascular disease, metabolic disorders, obesity, and osteoporosis. OA is independently associated with increased cardiovascular risk in both men and women; however, registry data suggest that this association may be stronger in women. For example, in a Danish national registry-based longitudinal cohort study, women (n = 1,838,434) with knee or hip OA had a 44% increased risk of developing cardiovascular disease compared with those matched women without OA (OR 1.44, 95% CI 1.43–1.46), whereas men demonstrate a smaller, though still elevated, increase in risk of 24% [192]. Although diabetes prevalence is generally higher in men than in women, evidence suggests that its relative impact—particularly in terms of cardiovascular and metabolic complications—may be greater in women, which may influence how patients are risk-assessed before surgery [193,194].

Obesity further contributes to adverse outcomes through both altered joint biomechanics and systemic inflammatory effects and is associated with increased complications and infection risk after hip and knee arthroplasty [195,196]. Osteoporosis affects around one in five women over 50 in the UK [197]. Reduced bone mineral density adversely affects bone quality and is associated with an increased risk of peri-operative complications, including a 1.5–2-fold-higher risk of intra-operative or periprosthetic fracture, as well as implant loosening and revision surgery [198,199]. The co-existence of obesity and osteoporosis may further compound risk, as patients may experience both increased mechanical loading from excess body weight and reduced skeletal integrity [196].

Efforts to model OA and MSK injury alongside co-morbidities in women remain challenging, as many epidemiological and health economic models continue to treat OA as an isolated condition. This reflects a broader limitation within MSK research, where sex-specific analyses are often under-reported or inconsistently incorporated, despite evidence that these factors influence disease pathways and outcomes [200]. Large-scale data resources, such as the National Joint Registry and UK Biobank (UKB), provide valuable insights into population health and disease patterns. A UKB study by Hollis et al. demonstrated that disease pathways differ by sex, with women at increased risk of developing OA following joint injury, highlighting the importance of accounting for sex-specific risk profiles in modelling [201]. Consistent with this, a recent UKB analysis demonstrated that female sex and older age at cartilage surgery are associated with increased progression to knee arthroplasty, with sex-specific differences in time to arthroplasty most apparent after debridement and microfracture [202]. Together, these findings emphasise the importance of accounting for sex-specific disease trajectories. However, while UKB provides valuable population-level insight into disease interrelationships, limitations in clinical detail restrict its ability to fully characterise surgical pathways and multimorbidity effects in women.

## 6. Gaps and Future Directions

Despite growing interest in the role of sex hormones in OA, clinical evidence remains limited. Although several studies have investigated hormonal approaches to OA management in women, encompassing preventive, pharmacological, and rehabilitative interventions, the available literature is characterised by a small number of trials, heterogeneous interventions, and a predominance of early-phase studies. Preclinical investigations are considerably more numerous and have produced promising findings supporting the potential role of hormonal modulation in OA management [203]. However, translation into clinical practice remains challenging due to the limited number of adequately powered randomised controlled trials. To date, clinical studies include a preventive trial in overweight and obese women, a feasibility study of HRT combined with bazedoxifene (a selective oestrogen receptor modulator) in postmenopausal women with hand OA, and a recent double-blind trial evaluating oestrogen replacement therapy combined with resistance exercise in older women with knee OA [150,204,205]. Additional rehabilitation-focused interventions, including muscular endurance, balance, and gait-training programmes, have also been evaluated in older women with medial knee OA [206]. Collectively, these studies suggest therapeutic potential but highlight the need for larger, robust trials with longer follow-up periods to determine efficacy, safety, and the patient populations that will gain benefit.

A key observation from the literature is that, although women constitute an adequate proportion of participants in OA clinical trials, older adults remain under-represented [207]. This further supports our earlier narrative indicating that sex is increasingly recognised as an important determinant of OA development, progression, and treatment outcomes rather than merely a confounding variable [208]. Despite this, hormonal status is rarely assessed using objective biochemical measures; instead, studies typically rely on descriptive classifications such as postmenopausal status without confirmation through serum or plasma hormone analyses. In addition, sex-specific subgroup analyses are often not predefined in trial protocols and, when performed, are frequently limited to post hoc endpoints. Collectively, these limitations restrict our understanding of the interplay between hormonal alterations and OA outcomes. The available evidence nevertheless suggests that sex-specific trial design, including objective assessment of hormonal status and adequately powered sex-stratified analyses, should be more prominently incorporated into contemporary OA research.

A further limitation of the current evidence is the predominance of knee-focused research. Knee, hip and hand OA represent distinct phenotypes with differing anatomical, biomechanical and risk-factor profiles. Future studies examining female-specific and hormonal influences should therefore report outcomes according to the joint site and OA phenotype rather than treating OA as a single outcome. Greater representation of hip and hand OA is particularly needed to determine which life-course mechanisms are shared across joints and which are site-specific.

There is currently an evidence gap relating to transgender and gender-diverse populations, who are largely absent from current OA research. Longitudinal studies examining musculoskeletal outcomes in individuals receiving gender-affirming hormone therapy may provide valuable insight into the effects of sustained changes in sex-hormone exposure on joint tissues, pain and OA risk.

Future research should move towards prospective, longitudinal cohort designs that recruit women before the menopausal transition and follow them through perimenopause into postmenopause. Such studies should incorporate repeated measurements of circulating sex hormones, including oestradiol, progesterone, FSH and other relevant endocrine markers, rather than relying solely on menopausal status or single-time-point measurements. Reproductive history should also be collected using standardised measures encompassing age at menarche and menopause, menstrual regularity, parity, breastfeeding exposure, contraceptive use and formulation, hormone replacement therapy, and natural, surgical or premature menopause. These data should be integrated with joint-specific assessment of OA using standardised imaging and biochemical markers of cartilage, bone and inflammatory activity, recognising that mechanisms and outcomes may differ between knee, hip, hand and post-traumatic OA. Longitudinal phenotyping should additionally incorporate biomechanical measures, including joint loading, alignment, muscle strength and physical activity, alongside metabolic factors such as body composition, adiposity, glycaemic status and systemic inflammation. Studies including both women and men should prespecify adequately powered sex-stratified analyses, while studies focused on women should account for reproductive stage and hormonal status within their analytical design. This integrated approach would allow temporal relationships between hormonal transitions, biomechanical and metabolic exposures, and subsequent OA onset and progression to be examined and may help identify critical periods during the female life course when OA risk is most amenable to modification.

## 7. General Discussion

This review on OA risk through the female life course was long overdue. The preclinical and clinical publications revealed a large body of evidence to support the narrative of increased OA risk across each life phase/transition in women. Female hormonal phases/transitions covered all the known risk factors that are associated with OA onset and progression, including injurious triggers, low-grade inflammation, altered metabolism and biomechanics. However, the relative importance of these mechanisms differs according to the joint site and OA phenotype, and much of the developmental, biomechanical and injury-related evidence identified in this review relates specifically to knee OA and knee PTOA. Associations between the female life course and OA risk are increasingly well documented; however, the causal pathways underlying these relationships remain unestablished. Reproductive milestones may act as markers of broader exposures rather than representing independent causal determinants of OA. How MSK injury and OA were experienced by women, in terms of pain perception, and the underlying mechanisms driving these processes are now well described and should be considered at each of the life phases/transition, although the pathways through which this occurs should not be assumed to operate equally across knee, hip, hand and PTOA.

It is encouraging that this research field, especially in more recent years, is receiving recognition and is being funded nationally and internationally to support the increasing body of multidisciplinary evidence. However, this has been a chronically neglected research area historically, and there remains a large quantity of additional research to undertake. A thorough understanding of sex-specific mechanisms at each life phase/transition is required, including greater clarification of which mechanisms are shared across OA phenotypes and which are joint-specific. Evidence relating to hip and hand OA remains less extensive than that available for knee OA, limiting the extent to which findings can currently be generalised across anatomical sites. How such mechanisms vary across genotypes and emerging OA endotypes also need to be more fully understood. Recognising and integrating female-specific OA risk across the life course is no longer simply an opportunity for discovery; it is a prerequisite for delivering equitable, effective, and precision-based OA prevention and care.

## Figures and Tables

**Table 3:** Summary of studies investigating the association between hormone replacement therapy (HRT) and osteoarthritis risk, symptoms, structural outcomes, and therapeutic potential.

HRT Related	Physiological Domain	Population	Study Design	Main Findings	Reference
OA Risk	Knee osteoarthritis risk	257,164 participants from 13 epidemiological studies	Meta-analysis	HRT use was associated with an increased risk of knee OA (HR 1.24, 95% CI 1.07–1.45). Current HRT users had a significantly increased risk, whereas past users showed no significant increase.	[124]
OA Risk	Knee OA and menopausal hormone therapy	Postmenopausal women	Observational study	Women receiving menopausal hormone therapy had a different prevalence/risk of knee OA compared with non-users, supporting an association between HRT exposure and knee OA.	[126]
OA Risk	Hand osteoarthritis risk	3440 hand OA cases and 13,760 matched controls (UK CPRD)	Nested case–control study	Women initiating HRT around menopause had a lower risk of hand OA (adjusted OR 0.72). The protective association disappeared after HRT cessation.	[118]
OA Risk	Spinal osteoarthritis	4265 Korean adults	Nationwide cross-sectional study	Women receiving HRT had a lower prevalence of spinal OA. Longer duration of HRT (>1 year) was associated with a greater reduction in spinal OA risk.	[119]
OA Risk	Hip OA	Elderly white women	Case–control study	Oestrogen replacement therapy was associated with a lower risk of symptomatic hip OA.	[148]
OA Risk	Hand and knee OA protection	Women from the Chingford Study	Population-based cohort study	Suggested a protective association between HRT and hand and knee OA, although the evidence was non-definitive.	[150]
OA Risk	Generalised osteoarthritis	475 women aged ≥50 years undergoing knee replacement	Cross-sectional study	HRT use was not associated with reduced odds of bilateral or generalised OA, suggesting no clear systemic protective effect against established OA.	[151]
Hormonal and Reproductive Factors	Hand OA and reproductive factors	348 Tasmanian women	Cross-sectional study	Shorter reproductive hormone exposure was associated with greater hand OA severity, supporting a hormonal influence on hand OA.	[131]
Hormonal and Reproductive Factors	Knee joint replacement	Norwegian women	Population-based cohort study	Age at menarche and reproductive history were associated with knee replacement risk for OA.	[49]
Hormonal and Reproductive Factors	Hand OA subtypes	Women with symptomatic hand OA	Retrospective study	Explored associations between menopause, HRT use and different hand OA phenotypes.	[152]
Structural Joint Effects	Cartilage morphology	Women aged 50–80 years	Cross-sectional MRI study	HRT was associated with greater cartilage volume and fewer cartilage defects.	[149]
Symptoms	Musculoskeletal pain	57 studies (3,958,702 participants)	Systematic review and meta-analysis	Up to 70% of menopausal women experience musculoskeletal pain. Evidence for HRT reducing general musculoskeletal pain remains inconsistent due to substantial heterogeneity between studies.	[128]
Symptoms	Hand osteoarthritis symptoms	711 menopausal women (238 painful hand OA, 240 quiescent OA, 233 controls)	Cross-sectional epidemiological study	No significant differences in hand OA symptom severity or disease activity were observed between women receiving HRT and those not receiving HRT.	[153]
Therapies	Symptomatic hand OA treatment	Postmenopausal women with symptomatic hand OA	Randomised placebo-controlled feasibility trial	Demonstrated that a randomised trial of conjugated oestrogens plus bazedoxifene was feasible and acceptable. Findings supported progression to a fully powered clinical trial but were not designed to demonstrate efficacy.	[129]
Therapies	Hand OA trial design	Postmenopausal women with symptomatic hand OA	Protocol for feasibility randomised placebo-controlled trial	Described the HOPE-e trial evaluating whether oestrogen-containing therapy improves pain and function in symptomatic hand OA, providing the methodological framework for the subsequent feasibility trial.	[130]

HRT, hormone replacement therapy; HOPE, Hand Osteoarthritis: Investigating Pain Effects of Oestrogen-containing therapy.

## Data Availability

No new data were created or analysed in this study. Data sharing is not applicable to this article.

## Abbreviations

The following abbreviations are used in this manuscript:

OA	Osteoarthritis
MSK	Musculoskeletal
ERα	Oestrogen Receptor Alpha
ERβ	Oestrogen Receptor Beta
RANKL	Receptor Activator of Nuclear Factor Kappa-B Ligand
OPG	Osteoprotegerin
Wnt	Wingless/Integrated Signalling Pathway
ACL	Anterior Cruciate Ligament
PTOA	Post-Traumatic Osteoarthritis
BMI	Body Mass Index
PMOS	Polyendocrine Metabolic Ovarian Syndrome
MRI	Magnetic Resonance Imaging
MOST	Multicentre Osteoarthritis Study
FSH	Follicle-Stimulating Hormone
HRT	Hormone Replacement Therapy
COCs	Combined Oral Contraceptives
POPs	Progestogen-Only Pills
E2	17β-Oestradiol
IL-6	Interleukin-6
TNF-α	Tumour Necrosis Factor Alpha
CRP	C-Reactive Protein
hs-CRP	High-Sensitivity C-Reactive Protein
IFN-γ	Interferon Gamma
IL-3	Interleukin-3
IL-8	Interleukin-8
IL-18	Interleukin-18
TNF-β	Tumour Necrosis Factor Beta
DMOADs	Disease-Modifying Osteoarthritis Drugs
NICE	National Institute for Health and Care Excellence
NSAIDs	Non-Steroidal Anti-Inflammatory Drugs
OARSI	Osteoarthritis Research Society International
EULAR	European Alliance of Associations for Rheumatology
UKB	UK Biobank
